# Nutrition Labeling to Prevent Obesity: Reviewing the Evidence from Europe

**DOI:** 10.1007/s13679-012-0020-0

**Published:** 2012-06-26

**Authors:** Stefan Storcksdieck genannt Bonsmann, Josephine M. Wills

**Affiliations:** European Food Information Council (EUFIC), Tassel House, Rue Paul Emile Janson 6, 1000 Brussels, Belgium

**Keywords:** Nutrition labeling, Obesity prevention, Consumer behavior, Public policy

## Abstract

Overweight and obesity are major public health problems in the European Union (EU). Providing nutrition information on foods and menus is considered a relevant means to guide consumers toward more healthful food choices, in part characterized by adequate energy intakes to achieve and maintain a healthy body weight. Various formats of back-of-pack and front-of-pack nutrition labeling can currently be found across the EU, with varying levels of penetration. Experimental studies show that consumers are reasonably able to understand and use the different systems to identify more healthful food products from given choice sets. However, European studies assessing the impact of nutrition labeling on actual dietary intake are scarce, and no real-life evidence exists linking nutrition label use with measured changes in body weight. This review summarizes how European consumers respond to nutrition labels when shopping for food or eating out of home, considering evidence published between 2007 and mid-March 2012.

## Introduction

Overweight and obesity are widespread in the European Union (EU). In its 2010 Implementation progress report about the “Strategy for Europe on Nutrition, Overweight and Obesity related health issues (2007–2013)”, the European Commission reports 30–70 % of EU adults to be overweight and 10–30 % obese [1]. As obesity is a condition of multifactorial origin, preventative measures need to consider various dimensions including individual, societal, economic, and environmental aspects. In this context, the “Strategy” identified consumer information as one of four priority areas for which it aims to provide guidance for action to EU Member States [1]. The 2004 Global Strategy on Diet, Physical Activity and Health by the World Health Organization listed nutrition labeling as an important means to meet the consumers’ requirement for “accurate, standardized and comprehensible information on the content of food items in order to make healthy choices” [2]. Likewise, the Organization for Economic Co-operation and Development (OECD) views nutrition labeling as “a main tool for preventing increasing rates of obesity and unhealthy diets in OECD countries” [3]. European public health professionals and other stakeholders appear to agree that (mandatory) nutrition labeling is one of the more important policy options for obesity prevention, but food and health education were also considered relevant [4]. This is supported by research showing that consumers report that they value the on-pack provision of nutrition information [5].

Although a new mandatory nutrition labeling legislation was adopted in December 2011 [6], any existing European studies into the role nutrition labeling could play in helping people choose healthful, balanced diets, were carried out on the backdrop of voluntary nutrition labeling. Only in the presence of a nutrition claim did nutrition labeling become mandatory in the EU, as laid down in Directive 90/496/EC of 1990 [7] and Directive 2000/13/EC of 2000 [8]. Depending on the claim, either the “big 4” (energy, protein, carbohydrate, and fat) or the “big 8” (“big 4” plus sugar, saturated fat, fiber, and sodium) had to be stated. An audit of the penetration of nutrition information on food and drink labels in the EU plus Turkey, carried out in 2008/2009, showed that the basic nutrition table was present (back-of-pack) on 84 % of over 37,000 products from five predefined food and drink categories (sweet biscuits, breakfast cereals, carbonated soft drinks, chilled fresh ready meals, yogurts) [9]. Penetration was lowest in Slovenia at 68 % and highest in the United Kingdom (UK) and Ireland at 97 %. There was an uneven split between “big 8” and “big 4” across countries, with the UK most often providing the “big 8” (94 % of all products audited), and Turkey least often (19 %). The average split was 49 % “big 8” and 34 % “big 4” (not summing up to 84 % due to rounding errors).

Dietary energy intake and physical activity are the most immediate contributors to energy balance. In case of sustained positive energy balance (ie, when more calories are being consumed than expended), overweight and obesity ensue. As energy (usually given in kJ and kcal per 100 g [mL]) is commonly one of the core information items in nutrition labeling, the above figures give an idea of the availability of dietary energy labeling on prepackaged food and drink products in Europe. Much less effort has gone into nutrition labeling outside the supermarket setting. In early 2006, McDonald’s introduced Guideline Daily Amount (GDA) labeling (energy, protein, fat, carbohydrates, and salt) on product packages in Italy in conjunction with the Olympic Winter Games held in Turin, followed by a rollout across Europe and the rest of the world in the months thereafter [10]. As concerns governmental action, the most prominent example appears to be the UK, where the national Department of Health in early 2011 initiated a Public Health Responsibility Deal [11]. Amongst others, the deal includes a pledge for out-of-home calorie labeling, “asking catering businesses, who sell food in out of home settings, to provide calorie information for customers on menus or menu boards, to help people make healthier choices” [11]. By early March 2012, 45 business partners had signed the pledge, 38 of them having submitted concrete delivery plans. First monitoring results were announced for April 2012, to be followed by annual reports every April thereafter.

Regardless of these efforts to make energy (calorie) information ubiquitous, the main question is whether people use this information when shopping for food or eating out, and with what outcome. Moreover, it is fair to assume that consumers need to know their energy requirements to make appropriate dietary choices based on the energy information provided. Two pan-European surveys [12, 13] indicated that while a majority of consumers know experts recommend to consume less calories, they were less certain about daily energy requirements for the average female (2000 kcal) and male (2,500 kcal). Knowledge about differences in calorie needs for men versus women and younger versus older adults was reasonably good, but over a third of respondents (over half in Poland) thought incorrectly children needed more calories than an adult man [13].

In their systematic review, Campos et al. [14] highlight that a number of studies have shown consumers to be struggling with quantitative nutrition label information. This was especially true for certain patient groups (diabetics, chronic kidney disease), older adults, adolescents, infrequent label users, and those with lower education levels, but appeared to be amenable to change through educational efforts targeted at label knowledge and understanding.

The need for simplicity was demonstrated by van Kleef et al. [15] who tested different front-of-pack energy signposts with consumer focus groups in the UK, Germany, The Netherlands, and France. Among the formats–from basic calorie labels to complex schemes including daily reference values and information about how much physical activity would balance out the stated calories–the simplest format was liked best. The more complex the scheme became, the less it was liked, and this effect was substantially more pronounced in German respondents compared to the other three countries.

To the best of our knowledge, research causally linking nutrition labeling with total energy intake over time, and corresponding changes in body weight, is lacking. A large part of the research into nutrition labeling stems from North America (the United States in particular), with comparatively little evidence from Europe [14]. The studies discussed below have looked at the potential of nutrition labeling to guide Europeans toward more healthful diets, mainly characterized by products lower in fat, saturated fat, sugar, or salt. As most of these key nutrients also provide calories, reduced dietary intakes thereof may be considered a proxy for lower energy intakes.

## Potential Impact of Nutrition Labeling on Food Purchases

### Modeling Approaches

Various studies [16–21] have modeled the potential impact of nutrition labels on dietary intakes or obesity in Europe. As an example, Sassi et al. [21] estimated that a mandatory nutrition labeling system for food sold in stores would decrease obesity rates in Europe by 2.5 % compared to a baseline situation where no such labeling existed. The model labeling scheme would inform consumers about nutrient content and portion size, supported by retailers posting explanations on how to read the labels and about the benefits of a healthy diet. Individual counseling by a combination of physicians and dietitians came out as most effective among the interventions assessed, with a population decrease in obesity prevalence of 6.5 %. Whereas the mandatory nutrition labeling intervention resulted in an estimated 15 million disability-adjusted life years (DALYs) averted, the individual counseling approach computed to a reduction of 50 million DALYs.

Modeling approaches are helpful to estimate what changes in consumers’ food choices (read: dietary intakes) are required to bring about significant public health effects. However, they provide little, if any, information about consumers’ actual use of nutrition labels, its determinants, and how label use could be improved to result in healthier food choices.

### Evidence from Retail Stores

In a nationally representative survey carried out with 11,781 respondents from France, Germany, Poland, Sweden, Hungary, and the UK, two thirds of shoppers were observed looking at the front of food and drink packages in the supermarket, with less than 15 % looking elsewhere on the pack [13]. Whereas many people tend to say they use nutrition information regularly when shopping for food [12, 22–24], only a relatively small number (16.8 %) was actually observed doing so [25••]. French consumers showed the lowest objectively measured use at 8.8 %, contrasted by UK consumers with 27.0 %. Similarly, the UK Food Standards Agency reported lower actual use than claimed by respondents [23]. Understanding and use of nutrition labels are positively correlated with female gender, higher income, better nutrition knowledge, and a general interest in healthy eating [14, 24, 25••, 26, 27].

Across surveys, calories and fat are stated fairly consistently as the most important nutrition information looked for, with the (back-of-pack) nutrition table being the main source [14, 24, 25••]. However, it was observed that few consumers look at the back of the pack [25••]. To help consumers access nutrition information more quickly, various front-of-pack schemes such as GDA and traffic light labeling have emerged in the EU over the past years, and used on a voluntary basis, yet with unclear outcomes [28]. The traffic light scheme, developed by the UK Food Standards Agency, depicts whether a food is high (red), medium (amber), or low (green) in key nutrients (eg, fat, saturates, sugar, and salt, and usually includes calories per portion [without color coding]). The GDA labeling system was developed in a collaborative effort by the UK government, consumer organizations, and the food industry, and shows the amount of key nutrients and energy per portion of a food or beverage, and what percentage that portion contributes to a person’s daily guideline amount of those nutrients or energy. Health logos such as the Choices logo or the Swedish Keyhole are a type of scheme that requires foods to fulfill certain nutrient profiling criteria before being eligible to carry the logo on pack. While these logos do not provide information about the energy content of foods and do not by default signal lower energy products, they guide consumers toward products with a lower content of certain calorific nutrients. Therefore, using more of these products instead of counterparts not eligible for the logo could be seen as a way of reducing energy intake.

GDA labeling appears to be the most widespread front-of-pack system, with an average EU penetration of 25 %, ranging from 63 % in the UK down to 2 % in Turkey [9]. As reported by Grunert and Wills [24], the UK retailer Tesco found that after introduction of GDA labeling, sales of some more healthful products went up whereas sales of comparable products with a less favorable nutrient profile went down. However, a number of methodological issues were pointed out, thus limiting the overall relevance of the data. Within the EU 7^th^ Framework Programme-funded project FLABEL (Food Labeling to Advance Better Education for Life) [29], a scientific assessment of the impact of introducing GDA labeling on product sales was attempted, again based on Tesco sales data [30]. Unfortunately, the analysis revealed that price changes at the same time as the GDA labeling introduction masked any potential effects the new labeling system may have had on sales.

As with Tesco, the UK retailer Sainsbury’s reported some beneficial effects on product sales in some categories from a nutritional perspective after the introduction of traffic light signposting on their own brand ranges (cited in [24]). However, when Sacks et al. [31•] studied sales data of ready meals and sandwiches from a UK retailer employing traffic light labeling, they found no consistent nutritionally desirable effects related to the introduction of the labeling scheme. In other words, consumers did not purchase more of the products nutritionally better for them in the presence of traffic light labeling. The authors concluded that larger studies of longer duration would be required before traffic light labeling could be considered a promising public health intervention.

Vyth et al. [32] aimed to study consumers’ use of the Choices logo in nine Dutch supermarkets and observed that shoppers who claimed to be logo users had purchased significantly more products bearing the Choices logo. Self-reported logo use was positively associated with several food choice motives including health, weight control, and product information. The authors qualified that 1) overall response rate was low, 2) not all eligible products carried the Choices logo (manufacturers voluntarily choose to join the scheme), and 3) observations were based on single shopping occasions, which may not reflect habitual food purchasing behavior.

Consumer preferences for a specific nutrition label format seem to differ by country [15, 27, 33]. However, regardless of whether consumers said or were objectively found to look at, like, understand, or use nutrition labeling systems, none of the above studies allows any conclusion as to whether consumers’ purchasing decisions were in any way influenced by the existence of such information. Findings from a study carried out in Germany and Belgium suggest that nutrition labeling has little impact on consumers’ buying decisions [33]. The relatively higher use and understanding of nutrition labeling systems by UK consumers indicates, however, that “intensive public debate on nutrition and labeling issues can indeed affect people’s thinking and behaviour” [25••]. This is supported by findings from Möser et al. [33] showing that GDA labeling was preferred in Belgium while traffic lights were preferred in Germany. Both countries have a similar availability of GDA labels [9], but in Germany consumer organizations, health professional associations, and insurance companies strongly supported the introduction of traffic lights, and the topic was widely covered in the media.

### Evidence from Out-Of-Home Eating Settings

Frequent out-of-home eating is associated with higher intakes of total energy and energy from fat [34•], and a higher risk of overweight and obesity [35]. Therefore, providing guidance to consumers toward making healthier food choices when eating out may be considered appropriate. Our literature search yielded four European studies published since 2007 assessing the impact of nutrition labeling on dietary intake in out-of-home eating settings [36–39].

Posting nutrition information–in the format of star ratings for most healthful choices–at the point-of-purchase in two Belgian university canteens failed to significantly improve meal choices by customers [36]. Better objective nutrition knowledge, stronger health and weight control motives, and a greater openness to change meal choices at baseline determined the best results. However, the authors pointed out that a generically more healthful meal supply might have made the intervention more effective.

In a Dutch cinema setting, Vermeer et al. [37] labeled soft drinks with portion size and GDA information (experimental setting) or just the volume in milliliters (control) on two consecutive evenings and compared customers’ portion decisions. Neither portion information nor GDA labeling led to significant changes compared to the control condition, yet the relatively small sample size of 101 subjects may have resulted in insufficient statistical power to detect meaningful differences. Experimental labels were noticed by 68.8 % of participants and control labels by 49.8 %, the difference again not being statistically significant.

Vyth et al. [38] tested whether introducing the nutrient profile-based Choices logo on products in Dutch worksite cafeterias improved customers’ food purchase decisions from a nutritional point of view. A comparison of food sales (sandwiches, soups, salads, snacks, and fruits) in 13 intervention cafeterias, where the logo was placed on eligible products, with 12 control cafeterias over a period of 3 weeks showed no impact of the intervention.

Qualitative research commissioned by the UK Food Standards Agency [39] assessed the impact on consumers of posting calorie information in catering outlets. Dimensions considered were visibility of the calorie label, its location, format, and availability, as well as consumer understanding and usage, including impact on food choices. Standing out from other information was shown to be important, and this was aided by sufficient label size, the use of a distinct color, and possibly a consistent label format and location–findings that are in line with FLABEL data reported by Bialkova and van Trijp [40•]. Advertising the existence of calorie labeling increased consumer awareness, and respondents stated that provision on all food items would help them to accurately judge calorie contents of full meals [39]. Unfortunately, at the time of this assessment, calorie labeling in catering outlets had been very new, which is why actual use was reportedly low.

## What is Required to Make Nutrition Labeling More Helpful and Relevant to European Consumers?

In general, the influence of nutrition labeling on food purchasing decisions is weak, especially when compared to other factors such as taste, price, use by-date, brand, convenience, and family preferences [22–24, 30, 41]. However, addressing a few barriers identified by FLABEL and other researchers could help optimize nutrition label use and thus its impact on dietary intakes.

While nutrition labels are already widely available [9], complete penetration on food and drinks products is considered helpful [30, 31•, 32, 33, 34•, 35–39]. Consistent label format and positioning emerged as important factors for easy and quick access [39, 40•]. A previous representative survey involving six European countries showed that consumers can use different labeling systems similarly successfully to identify the most healthful option out of a choice of three ready meals/pizzas [25••]. This and other research [22, 23, 26] suggest it does not matter so much which system is used on product packages, as long as it is presented in the same format and place on all products. The provision of multiple systems should be avoided as it may cause consumer confusion and frustration [23, 42]. Consistency, especially if supported by promotional and education campaigns, should aid familiarity with the nutrition labeling system, which in turn may enhance actual use [43].

Consumers’ attention and motivation remain major barriers to using nutrition labels [25••, 44•], thus limiting any potential impact on health. Eye-tracking research measuring how long consumers look at nutrition labels indicated a time span of 25–100 ms regardless of the system used [30]. This period is far too short for any conscious processing of the information. However, the presence of a health logo can slightly improve attention to the nutrition label [40•], which may be considered relevant within the (fairly) common condition of shopping under time pressure.

Actively seeking out nutrition information requires a certain level of motivation. Such motivation could derive from the presence of a diet-related disease (eg, type 2 diabetes, hypertension), which would make nutrition information more personally relevant. Research shows that consumers with a health goal in mind are more likely to pay attention to and use nutrition labels [32, 44•, 45]. The opposite was observed when consumers followed their own preferences or were given a hedonic goal.

## Beyond the Nutrition Label

Low income and lack of time may be major barriers to buying more basic and healthful foods; providing more information–in the form of nutrition labeling–will increase neither of these two [14, 24, 46]. Furthermore, nutrition labels are more likely to be read by those who have an interest in healthy eating, show better nutrition knowledge, and thus may display healthier eating patterns already [14, 25••]. In this context, findings from FLABEL [30] and others [32] indicate that expanding a given food/drink category by adding more healthful products can improve overall healthfulness of actual choice by the consumer. Nutrition labels, especially health logos, are seen as a potential driver for product reformulation in an attempt to meet eligibility criteria [17, 26, 30, 47]. Furthermore, Barreiro-Hurlé et al. [48] noted that clear and truthful nutrition and health claims may reach out to those who are less likely to read nutrition labels, such as people with lower nutrition knowledge or more hedonic lifestyles.

## Conclusions

Nutrition labeling is considered a relevant component of public health policies attempting to stem the obesity epidemic in Europe. Scientific evidence to prove its actual use by consumers and the resulting impact on dietary energy intake, body weight, and health remains largely absent. Allison [49••] rightfully stated that “[i]f we are to understand the value of any macro-environmental manipulation intended to reduce obesity levels, we must eventually measure body weight, fat, or obesity levels”.

While consumers like to see nutrition information on food and drink packages and appear able to use any labeling scheme to choose more healthful options out of a limited choice set under experimental conditions, they pay little attention to nutrition labels in real life. This lack of attention is partly driven by a lack of motivation, but the grander scheme suggests that price, taste, convenience, and shopping habits are simply far more important than nutrition information when making food purchasing decisions. Shopping under time pressure–a common phenomenon among today’s consumers–further impedes nutrition label use for healthy food shopping.

The new EU food information regulation, which makes nutrition labeling mandatory, provides an opportunity for monitoring the impact of this policy on public health. However, simply providing such information will not be enough to justify expectations for a (positive) change in people’s dietary habits. Instructive educational campaigns are required that raise awareness, understanding, and the motivation to use nutrition labels, taking into account the diverse needs of the European consumers.

## Authors’ Note

After several years of negotiation, the European Commission in December 2011 made nutrition labeling mandatory on food and drink products. With a few exemptions, manufacturers must disclose information on the package about energy and six nutrients; total fat, saturated fat (saturates), carbohydrates, sugars, protein, and salt–in this order, and expressed per 100 g (mL) of product [6]. This information should be presented in the same field of vision, usually on the back of the pack, and may in addition be expressed on a per portion basis. Manufacturers who already provided nutrition information in the past must comply with the new regulation by December 2014, whereas those who have yet to introduce nutrition labeling on their products are given until December 2016.

Front-of-pack labeling remains voluntary under the new regulation, yet if information is repeated on the front of the pack, specific rules apply. Front-of-pack information can be the content of energy alone or in combination with fat, saturates, sugar, and salt. Energy can be presented per 100 g (mL) alone or additionally expressed per portion. The new regulation maintains the requirement to display energy in both kilojoules (kJ) and kilocalories (kcal) (there are 4.2 kJ in each kcal). When this information is declared for a portion or unit (eg, amount per biscuit), the size of a portion/unit must also be indicated, in conjunction with the number of portions or units contained in the package.
